# How to Receive More Funding for Your Research? Get Connected to the Right People!

**DOI:** 10.1371/journal.pone.0133061

**Published:** 2015-07-29

**Authors:** Ashkan Ebadi, Andrea Schiffauerova

**Affiliations:** 1 Concordia Institute for Information Systems Engineering (CIISE), Concordia University, Montreal, Quebec, Canada; 2 Department of Engineering Systems and Management, Masdar Institute of Science and Technology, Abu Dhabi, United Arab Emirates; Institute of Tropical Medicine (NEKKEN), Nagasaki University, JAPAN

## Abstract

Funding has been viewed in the literature as one of the main determinants of scientific activities. Also, at an individual level, securing funding is one of the most important factors for a researcher, enabling him/her to carry out research projects. However, not everyone is successful in obtaining the necessary funds. The main objective of this work is to measure the effect of several important factors such as past productivity, scientific collaboration or career age of researchers, on the amount of funding that is allocated to them. For this purpose, the paper estimates a temporal non-linear multiple regression model. According to the results, although past productivity of researchers positively affects the funding level, our findings highlight the significant role of networking and collaboration. It was observed that being a member of large scientific teams and getting connected to productive researchers who have also a good control over the collaboration network and the flow of information can increase the chances for securing more money. In fact, our results show that in the quest for the research money it is more important how researchers build their collaboration network than what publications they produce and whether they are cited.

## Introduction

About 100 years ago, power and wealth of the nations were measured by their amount of natural resources or the industrialization stage. Apart from the human capital that is an essential factor for scientific development and innovation [1], knowledge has also become a new worthy capital and a basis for competitiveness [2]. In this respect, it is essential to strive to increase the production of the knowledge, which could be estimated by the research outcomes in terms of publications and scientific applications [3]. Funding has been acknowledged as one of the main drivers of scientific activities [4]. It can play a significant role in defining new scientific projects and/or setting priorities on the existing projects.

Investment strategies on research and development (R&D) can affect the performance of the funded researchers and their interactions with other scientists. In addition, funding can influence the size and efficiency of R&D sector and its productivity [5]. Higher scientific performance can be reached by better funding allocation through selecting highly prolific research groups or well-defined projects, supporting novel ideas, and targeting structural changes such as promoting scientific collaboration networks [6]. However, different nations follow various research patterns and their institutional and economic structures greatly differ. Hence, the composition of the budget which different countries are allocating to R&D varies as well. As a result, various allocation patterns are used worldwide to distribute the research funding among the universities and research institutes [7].

Governments put significant efforts in defining and executing systematic procedures for evaluating the performance of researchers in order to be able to better allocate research funds among them. In addition, policies on the R&D activities have evolved over the past fifty years [8–9]. Beginning with the research promotion through public research centers, motivating and incentive mechanisms were introduced during 1960s and 1970s, first by the *research councils* [10] and later through *strategic R&D programs* [11], in order to further stimulate firms and universities to advance their scientific activities. Nowadays, different countries are experiencing various types of governmental interventions and policies. Due to the importance of scientific development and the limited financial resources assessing the effectiveness of government policies as well as the performance of the funded researchers is becoming more vital.

In Canada the importance of receiving research funding is on the rise especially among the academic researchers [12], which could make the competition for getting more grants very tight. The Canadian Institute of Health Research (CIHR), the Natural Sciences and Engineering Research Council (NSERC), and the Social Sciences and Humanities Research Council of Canada (SSHRC) are the three main funding bodies operating in Canada. CIHR, established in 2000, is responsible for the support of health research in Canada. NSERC, established in 1978, is the main federal agency of the country funding natural sciences and engineering research, and SSHRC, also established in 1978, supports a wide range of research in social sciences and humanities. The Canadian government (like most governments in Western countries [13]) has focused on the universities as the key research units of the country over the past 25 years in order to secure the national competitiveness worldwide [12]. Therefore, several policies have been set (*e*.*g*. commercialization of university research, setting research priorities and promoting targeted areas) in order to foster university-industry collaboration, to encourage academic researchers’ scientific activities and to better establish the key role of universities [14]. Changes in federal funding policies, lack of university operating budgets, higher priority of the selected strategic research projects, and rising research costs have made the research grants more important than ever to the Canadian researchers [12].

A lot of studies have analyzed the impact of funding on the productivity and performance of the funded researchers in terms of quantity and quality of their publications at micro (*e*.*g*. [5,15–17]) or macro level (*e*.*g*. [7,18]). In addition, there exist some studies that focused on the scientific collaboration among researchers and that assessed the impact of funding on the formation or rate of collaboration (*e*.*g*. [19–22]). However, according to our knowledge there is no study that explores the factors which can influence the amount of funding that researchers receive. Such study can bring two main benefits to the scientific community: First, using various allocation strategies (*e*.*g*. performance based or educational size based) governments are devoting a considerable part of the budget to research annually. The proposed analysis can shed a light on the results of the funding allocation policies. In other words, we identify the true factors that seem to actually play a role in the decision making of the committees allocating money to the researchers. Moreover, by focusing on the identified significant factors researchers can use this study as a guide in their efforts to secure more research money in future. Second, it is often heard in the scientific communities that the rich get richer! This is known in sociology as the Matthew effect which means that higher amount of money available to a researcher brings him/her some advantages that will result in even more research money for the researchers in future [23]. But what is it exactly which makes the rich become richer? Apart from the factors directly related to the research effort (*e*.*g*. past productivity and better access to the required equipment), we assume that social factors (*e*.*g*. professional relations, collaboration) also play a role in the research funding success. Hence, this analysis involves not only the evaluation of the bibliographic scientific factors but also the assessment of the impact which the collaboration patterns among researchers have on their subsequent funding level.

This study considers funding as a dependent variable and systematically analyzes the impact of several determinant factors such as past productivity of researchers, collaboration network variables or career age on funding the researchers obtain. Therefore, this paper contributes to the existing literature in two ways. First, to our knowledge, no study has identified and examined the factors which determine the allocated funding to the researchers at the individual level. We will address this gap through employing statistical analysis techniques on an extensive dataset. Second, we will identify the profile of highly funded researchers and thereby will shed a light on how a researcher can obtain more funding. Our basic motivating questions in this research are: What factors are important in getting more funding? What the profile of the highly funded researchers looks like? Is funding biased towards senior researchers? The remainder of the paper proceeds as follows: next section presents the data and methodology; the empirical results and interpretations are followed next; Section “Conclusions” presents the findings of the research; and the limitations and some future research directions are discussed in “Limitations and Future Work” section.

## Data and Methodology

### Data

The data for this research was gathered in four phases. In the first phase, we searched for the data on funding received by Canadian researchers. Due to various limitations we decided to focus only on researchers in natural sciences and engineering. We extracted the funded researchers’ data from The Natural Sciences and Engineering Research Council of Canada (NSERC). NSERC is the main federal funding organization in Canada and almost all the Canadian researchers in natural sciences and engineering receive a research grant from NSERC [16]. Moreover, the NSERC funding database is public and freely available.

In the second phase, after collecting the funded researchers’ data, Elsevier’s Scopus was considered for gathering all the information related to the articles (*e*.*g*. co-authors, their affiliations, year of publication, citations) that were published by the funded researchers within the period of 1996 to 2010. We decided to focus on the period of 1996 to 2010 since the data coverage of Scopus was better after 1996.

The third phase involved collection of the data on the quality of journals. We used SCImago to collect the impact factor information of journals in which the articles were published for the period of 1996 to 2010. SCImago was chosen for two main reasons. First, it provides annual data of the journal impact factors that enables us to perform a more accurate analysis since we are considering the impact factor of the journal in the year that an article was published not its impact in the current year. Secondly, SCImago is powered by Scopus that makes it more compatible with our articles database.

All the collected data were integrated into a MySQL database. In the last phase of the data gathering procedure, we used Pajek software to construct the co-authorship networks of the funded researchers and to calculate the network structure variables at the individual level and in a three year time window. The calculated network structure indicators were also integrated into the database. The final database contains 174,773 records of the funded researchers. In the next section, we discuss the methodologies used in this research.

### Methodology

Funding is more often allocated to a team of researchers rather than a single researcher [24]. Limited financial resources made it impossible to allocate the required research support to all the researchers who ask for it. This resulted in a pooled resource allocation, where researchers are forced to collaborate together more [25]. Through collaboration researchers can get access to precious external resources that might enable them to secure greater amounts of money for their own research. Moreover, as the nature of the modern science has become more interdisciplinary, the need for complementary knowledge has made the collaboration among researchers from various scientific fields and disciplines essential. Selecting the right collaborators can be critical for the success of such research projects, and knowing the right people in the researcher’s professional network can have a great effect on his/her future career. It is thus expected that the collaboration patterns and positions of the individual researchers within their collaboration networks can influence their success in receiving future research grants. To account for the impact of the scientific collaboration, we employed social network analysis, constructed the co-authorship network of the researchers and measured the structural network properties. Despite some drawbacks (*e*.*g*. [25–26]), co-authorship is considered as the common way of measuring research collaboration in the literature [27–28].

Hence, in this study co-authoring an article was assumed as a sign of collaboration among researchers. We had no information on the length of the collaboration among researchers. Although various time lengths have been considered for collaborative networks, three (*e*.*g*. [24,29]) and five year (*e*.*g*. [30–31]) time windows are the most common in the literature. We considered both three and five-year time windows and found that the results are more significant, robust and meaningful for the three-year time window. One reason could be the number of time intervals in our study as they drop if we consider the five-year time window. Therefore, the three-year time window was considered for the life of each created collaboration link in our network and this time window was shifted forward from the funding year of 1999 to 2010. For example, for a researcher who was funded in 1999 we constructed his/her co-authorship networks during the period of 1996 to 1998 and calculated the network structure indicators for the mentioned three-year sub-networks. More specifically, we calculated four network structure variables that are “*betweenness centrality*”, “*degree centrality*”, “*eigenvector centrality*”, and “*clustering coefficient*” and assessed their impact on funding. The definition of the mentioned network variables are as follows:

Betweenness centrality (*bc*) focuses on the role of intermediary individuals in a network. The betweenness centrality of a node *k* is measured based on the share of times that a node *i* reaches a node *j* via the shortest path passing from the node *k* [32]. In our co-authorship network, the more often a researcher lies on the shortest path between two other researchers in the network, the higher betweenness centrality it has. High betweenness centrality of a researcher thus indicates the high control of that researcher over the information which will reach other researchers in the network [33]. This measure allows us to identify *gatekeepers*, influential researchers who are not only able to affect the flow of knowledge through the network but also to bridge different communities/researchers. We expect that such researchers might benefit from their control over the network to gain higher level of funding. Theoretically, betweenness centrality of node *k* (*bc*_*k*_) is defined as follows:

$$bc_{k}=\sum_{{}_{i\neq k\neq j}}\frac{\sigma_{ij}\left(k\right)}{\sigma_{ij}}$$

where *σ*_*ij*_ is the total number of shortest paths from node *i* to j and *σ*_*ij*_ *(k)* is the number of shortest paths from node *i* to node *j* that contains node *k*.

Degree centrality (*dc*) is defined based on the number of ties that a node has (*i*.*e*. degree of the node) in an undirected graph. Researchers with higher degree centrality are expected to be more active since they have higher number of ties to other researchers [33]. Having higher number of direct connections might enable a researcher to work in a larger team and get access to other external resources (*e*.*g*. financial resources, new connections, new projects) that might result in securing more money. Degree centrality for the node *i* is defined based on the node’s degree and then the values are normalized between 0 and 1 to be able to compare centralities:

$$dc_{i}=\frac{degree_{i}}{highest\;degree\;in\;the\;network}$$

Eigenvector centrality (*ec*) is based on the idea that the importance of a node in the network depends also on the importance of that node’s neighbors. Hence, in our co-authorship network a researcher has a high eigenvector centrality if he/she is connected with other scientists who are themselves important players in the network. In other words, eigenvector centrality measures how well connected a researcher is in the network. According to Bonacich [34] the centrality of a node is defined based on sum of its adjacent centralities. In our network, we name researchers who have high eigenvector centrality values as *diplomats* and we expect them to be able to bridge connections, shape collaborations among researchers and play an important role in setting priorities on scientific projects.

The last network structure variable whose impact on funding we evaluated is clustering coefficient (*cc*). This measure is also called *cliquishness* in the literature and it is defined as the likelihood that two neighbors of a node are also connected to each other [35]. Watz and Strogatz [36] define clustering coefficient based on a Local Clustering Coefficient (*lcc*) for each node within a network. The definition of *lcc* is:

$$lcc_{i}=\frac{number\;of\;triangles\;connected\;to\;node\;i}{number\;of\;triples\;centered\;on\;node\;i}$$

The denominator of the above formula counts the number of sets of two edges that are connected to the node *i*. The overall clustering coefficient is calculated by taking average of the local clustering coefficient of all the nodes within the network. Hence,

cc=1n∑i=1nLCCi

in which *n* denotes the number of vertices in the network. This measure returns a value between 0 and 1 in a way that it gets closer to 1 as the network interconnectivity increases (higher cliquishness). Researchers with high cliquishness tend to cluster with other researchers resulting in tightly knit group collaboration with high number of connections among the team members. Therefore, analyzing the impact of cliquishness on funding can help to identify whether the density of collaboration among researchers also affects their success in obtaining research grants.

Apart from the network structure variables that represent several aspects of scientific collaboration among the researchers, the measures directly related to the scientific efforts of researchers were also calculated and integrated into the statistical model as independent variables. In order to assess the value of the research output we considered two proxies for the quality of the papers, one is based on the citation counts while the other one is based on the impact factor of the journals in which the articles were published. Both of them can serve as a proxy for quality, but with a slightly different meaning. High impact factor is generally accepted as a sign of the respectability of a journal among scientists, and as such it can be used as an indicator of the quality and the level of contribution of the published articles as perceived by their authors and by their reviewers. The citations, on the other hand, are an indicator of the impact the article had on the research community. Since both proxies have some flaws, we decided to include both of them. *avgIf3*_*i-1*_ is calculated based on the average impact factor of the journals in which the author has published his/her articles within a three year time interval, and *avgCit3*_*i-1*_ is the average number of citations for the articles in the past three years.

Older researchers in general can be more productive [37–38]. Several factors like better access to the funding and expertise sources, more established collaboration network, better access to modern equipment, *etc*. may contribute to their higher productivity. Hence as a proxy for the career age of the researchers, we included a control variable named *carAge*_*i*_ representing the time difference between the date of the first article of a researcher in the database and the given year.

We also wanted to include the past funding of the researchers in the model, however, it was omitted due to high correlation between the past funding and the current funding. But to partially reflect the impact of past funding, the past productivity of researchers was included. As discussed earlier, it is expected that researchers with high amount of money in year *i* on average produce more publications that will bring them higher amount of money in year *i+1* (Matthew effect). Hence, past productivity was included to account for this effect. It is represented by *noArt3*_*i-1*_ in the model and is measured as the number of articles for a researcher in a three year time window. The reduced form of the regression model is shown in Eq (1).

$$fund_{i}=\;f\left(avgIf3_{i-1}\;,\;avgCit3_{i-1},\;noArt3_{i-1}\;,\;bc3_{i-1}\;,\;dc3_{i-1}\;,\;cc3_{i-1}\;,\;ec3_{i-1}\;,\;carAge_{i}\;,\;d_{i}\right)$$ (1)

We also used three types of dummy variables in our regression model that are represented in general by *d*_*i*_ in Eq (1). The first dummy variable indicates whether the researcher is in the largest component (largest connected sub-network) of the collaboration network. It is expected that researchers in the largest component can get in contact with other researchers easier than the ones in smaller components. This might help them to benefit from some external resources to secure more money. The second dummy variable indicates whether researcher is affiliated with industrial or academic environments. This indicator is based on the institution each of the researchers is affiliated with when publishing each of the papers. The last dummy variable evaluates the impact of being located in different Canadian provinces on securing more funding. STATA 12 software package was used to perform the statistical analysis. In the next section we discuss the results of the analysis.

## Results

### Descriptive analysis

**Fig 1:**
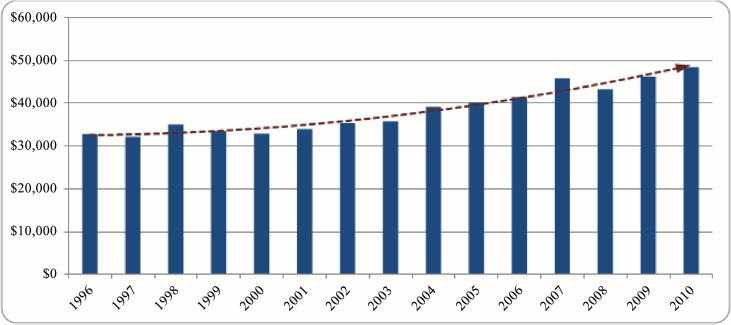
Average funding per distinct researcher, 1996 to 2010.

Before presenting results of the statistical analysis, we first examine the trends of some related indicators to provide a general picture. Fig 1 shows the average amount of NSERC funding granted to distinct individual researchers from 1996 to 2010. As indicated by the red dashed line in the figure average funding has followed an increasing trend during the examined time interval reaching from the level of $32,000 in the first considered year to around $49,000 in the final period.

**Fig 2:**
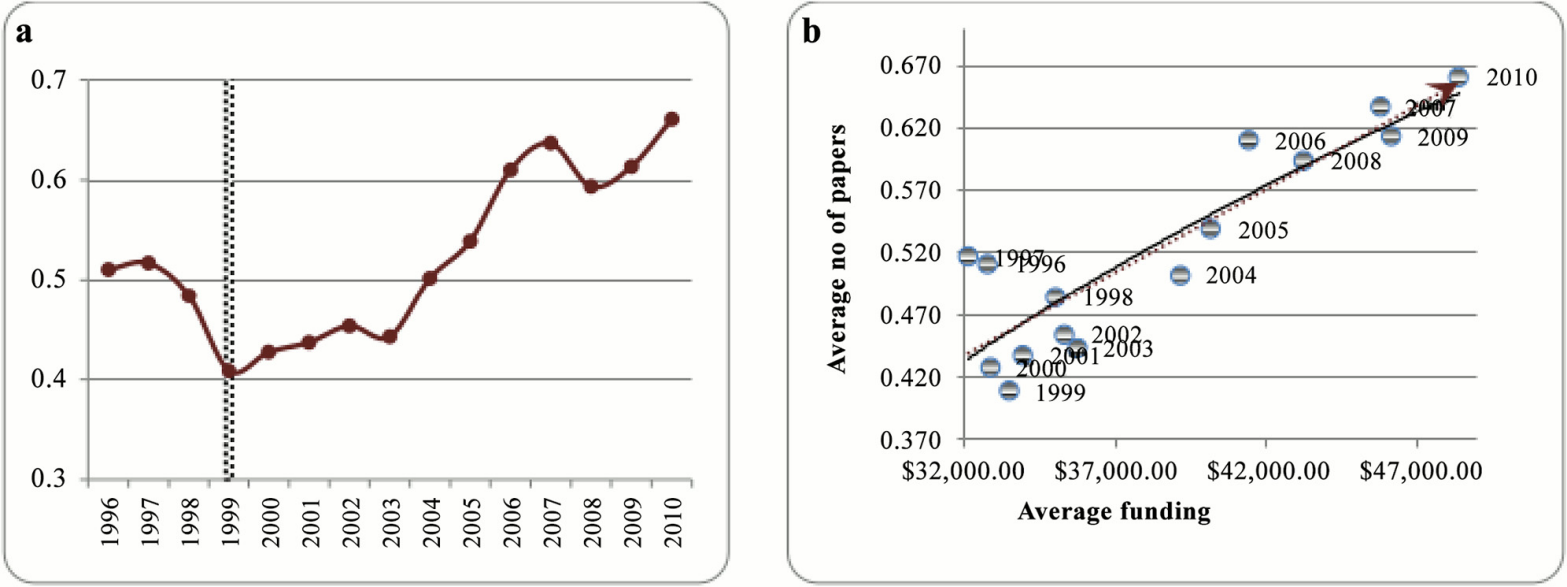
a) Average number of papers per researcher, 1996 to 2010, b) Average number of papers versus average funding, 1996 to 2010.

Researchers publish their results in books and journal articles, and present them in scientific conferences in order to ensure priority for their discoveries and raise their scientific reputation. Number of publications has been widely used in the literature as a proxy for scientific output. Fig 2a depicts the average number of papers per researcher during the examined time interval. The trend can be divided into two parts as indicated by the vertical dashed line in the figure that are: decreasing trend from 1996 to 1999 and increasing trend afterwards. The slope of the increasing trend becomes steeper after 2003 and it continues till 2007 while after a sudden drop in 2008 it continues to augment with almost similar slope. Fig 2b shows the overall relation between the amount of average funding and the number of publications (in the absence of other factors). Intuitively it seems that there is a positive relation between funding and scientific output.

**Fig 3:**
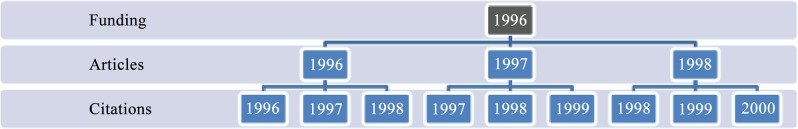
Example of the procedure for counting the citations received by the articles.

Apart from the rate of publications we have also analyzed the trend in their quality. As mentioned earlier, number of citations received by an article and the impact factor of the journal in which the article is published are the two most common measures for the quality of the paper. However, it is argued that journal impact factor cannot be considered as a good paper quality measure since it is highly discipline dependent and editorial policies can also affect the impact factor [39–40]. Number of citations has also some drawbacks (*e*.*g*. negative citations and self-citation) but citation based indicators are considered as the common practice in measuring the overall impact of an article [41]. We defined a three year time window for both funding and articles to calculate the average amount of citations. For example as it can be seen in Fig 3, for the funding year of 1996 we collected all the articles of the funded researchers for the period of 1996 to 1998. Then, we defined a three-year citation window for each of the publication years. In other words, we counted the citations for the period of 1996 to 1998 for the articles that were published in 1996, and from 1997 to 1999 for the articles published in 1997, and from 1998 to 2000 for the articles published in 1998. We followed the same procedure for the other funding years and in order to make a fair indicator we stopped at the funding year of 2008 since we had the publications for the period of 1996 to 2010 and the citations for the period of 1996 to 2012.

**Fig 4:**
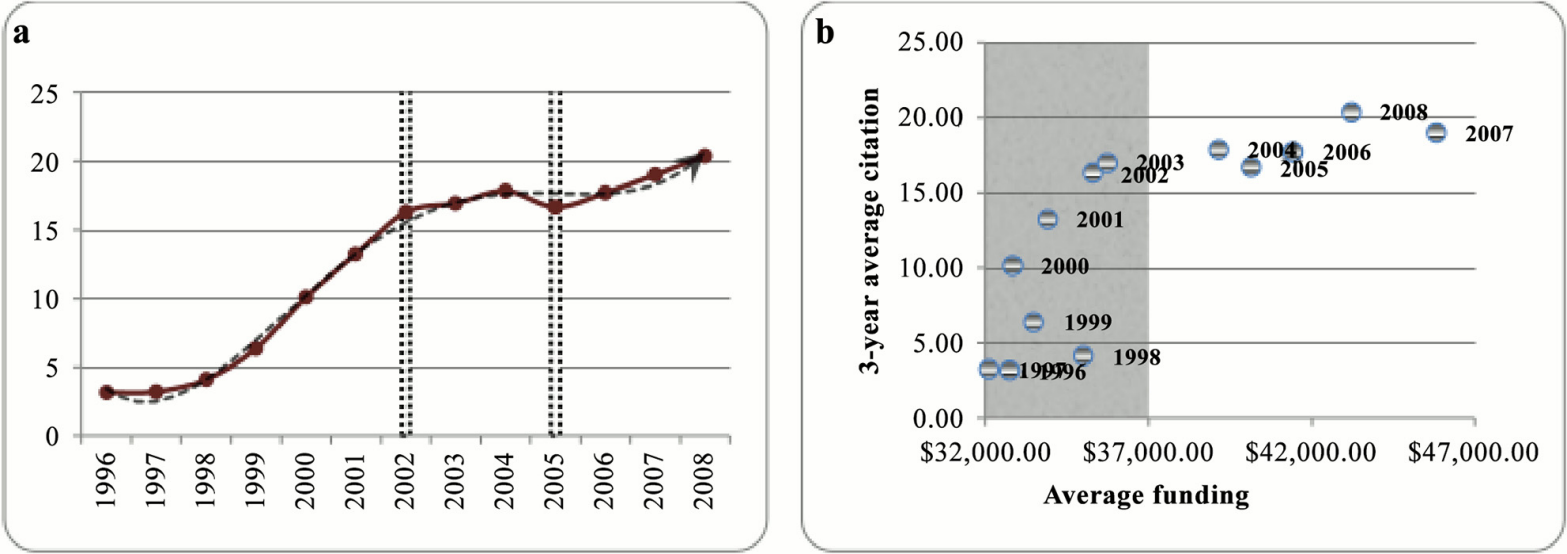
a) 3-year average citation counts, 1996 to 2008, b) Normalized 3-year average citation counts versus normalized average funding, 1996 to 2008.

Fig 4a depicts the trend of 3-year average citation indicator over the period of 1996 to 2008. The overall trend follows an increasing polynomial curve of degree 4. As indicated by the dashed vertical lines, the trend can be divided into three regions. Except for the period of 2002 to 2005 for which we see an almost steady trend, in the other parts the average number of citations has increased. The slope is much steeper for the period of 1998 to 2002. Fig 4b shows the average citations received by the articles versus the average amount of funding allocated to the researchers labeled for different years. As it can be seen, it seems that no relation exists between funding and quality of the papers. Specifically for the period of 1996 to 2003 that is shaded in Fig 4b, although the annual average amounts of funding are comparable (see only a very slight increase in Fig 1) a considerable difference is seen in the amounts of citations. This preliminary result is quite in line with Fortin and Currie [42] who focused on three scientific disciplines and found a weak relation between the amount of NSERC funding allocated to an individual researcher and the output quality. Of course this is a preliminary observation at the aggregate level as we just focused on average annual funding and 3-year average number of citations. We will further investigate this issue by incorporating various variables of different types and performing statistical analysis at the individual level of researchers.

**Fig 5:**
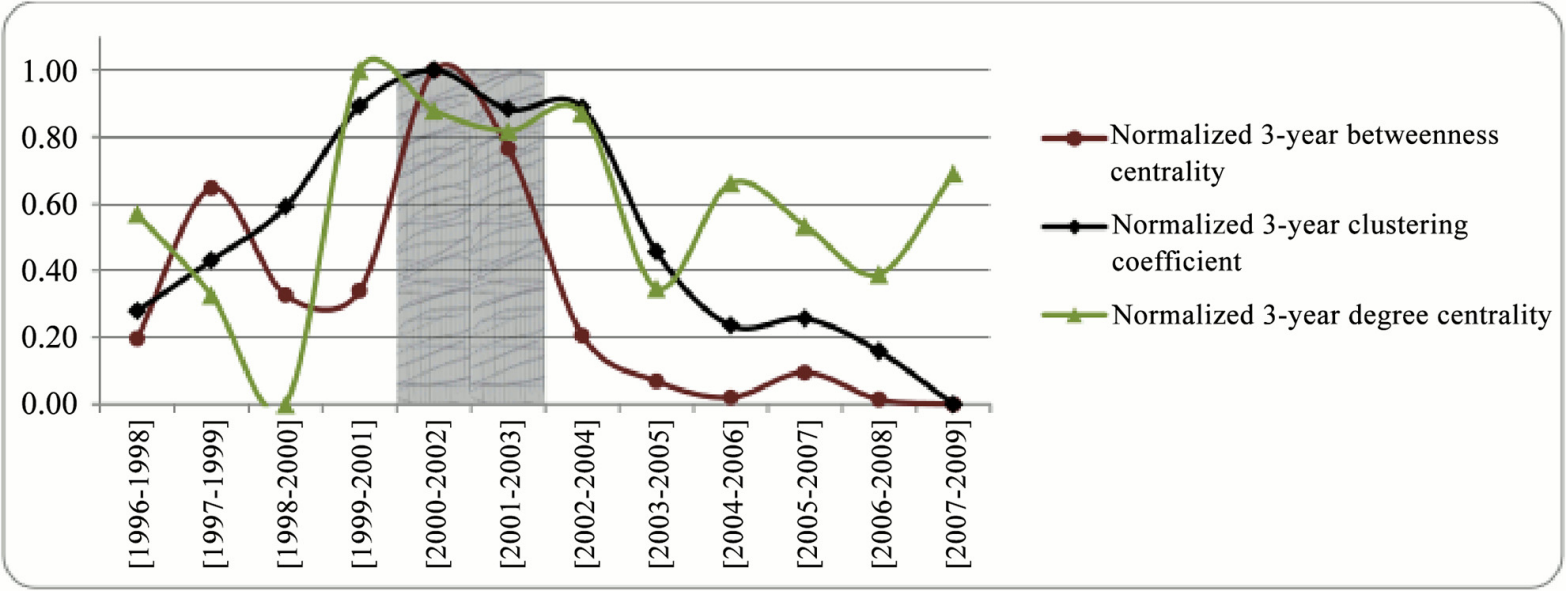
Network structure variables at the aggregate level.

Analyzing the trend of network structure variables in a three-year time window at the aggregate level reveals that within the period of [2000–2002] till [2001–2003] all the examined network variables were relatively high (the shaded area in Fig 5). Hence, it seems that no relation exists between the network variables at the aggregate level and the amount of average funding as the trend of funding has been slightly increasing during the whole examined period (Fig 1). One reason for the drop in the values of the aggregate network variables in the recent years can be the increasing trend of involvement of new researches in the network. We will investigate the impact of network variables more accurately by calculating them at the individual level and assessing their effect statistically in section 3.2.

**Fig 6:**
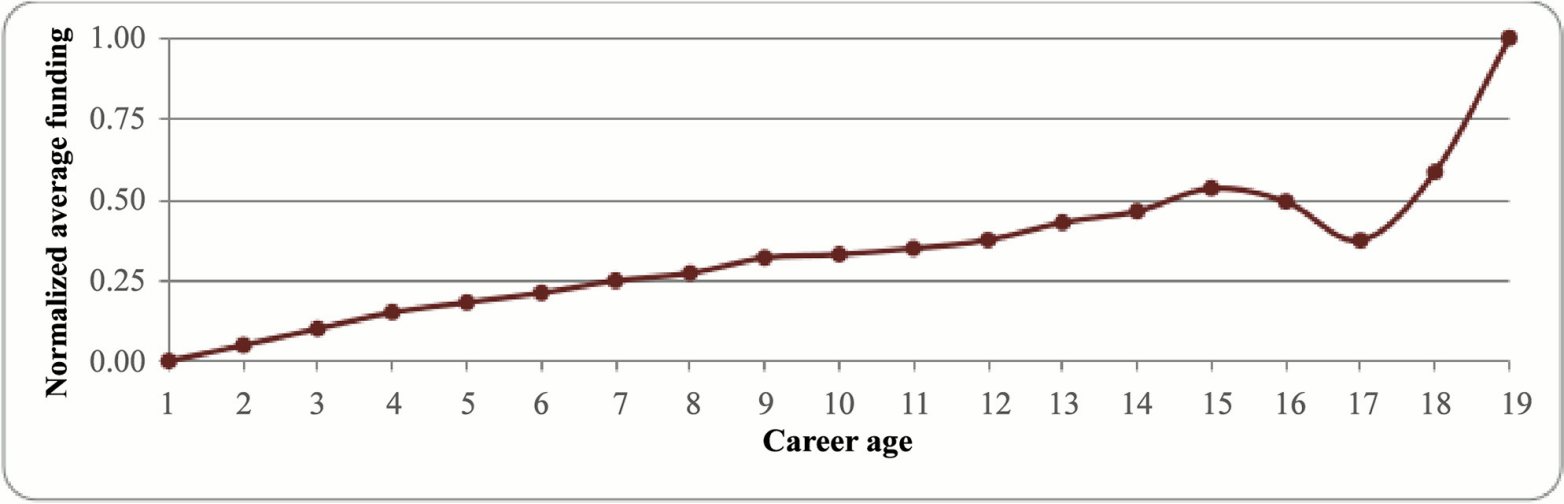
Normalized average funding per distinct researcher versus career age.

We also examined the interaction of the career age of scientists with the average amount of funding allocated to them. For this purpose, we searched over our publications database from 1992 to 2010 for each of the scientists in the database and set career age of a researcher to one on the date that he/she produced his/her first publication. Hence, for the period of 1992 to 2010 the career age ranges from 1 to 19. Having set the career age of the researchers, we focused on the range of 1996 to 2010 and compared the amount of funding allocated to the researchers of different career ages. According to Fig 6, it can be said that there is a positive relation between the career age of the researchers and the amount of funding that they have received until the career age of 15. However, some fluctuations are observed after the career age passes 15 reaching to a maximum at the age of 19. Hence, it seems that as researchers start their career the funding allocated to them is minimal at first, but continues to increase and reaches very high towards the end of their career. We will assess the impact of the career age of scientists on funding in the statistical analysis more accurately.

### Statistical analysis

As explained in section 2.2. four network structure variables (*i*.*e*. betweenness centrality (*bc*), clustering coefficient (*cc*), eigenvector centrality (*ec*), and degree centrality (*dc*)) along with two measures for the quality of the papers (based on journal impact factor (*avgIf*) and citations counts (*avgCit*)), as well as the number of publications (*noArt*) and the career age of the researchers (*carAge*, *control variable*) are considered as the independent variables. As it was observed in Fig 6, it seems that the relation between the career age and funding is non-linear, hence, in order to see the curvature of the career age impact the quadratic term was also included in the model. We considered all the researchers who are affiliated with universities, research institutes, and industrial firms and perform multiple regression analysis at the individual level. Moreover, we filtered the data to include only the researchers for whom all the network structure variables could have been calculatedas some network structure variables are sparser at the individual level (*e*.*g*. betweenness centrality). The reason is that only a limited number of researchers has a control over the network, *i*.*e*. betweenness centrality higher than zero. However, on the other hand the degree centrality is greater than zero for all the non-isolated nodes. This filtration resulted in 72,267 records of funded researchers.

Before running the regression model, we first analyzed the associations between dependent and independent variables. According to Table 1, the absolute value of all the correlation coefficients is lower than 0.46, which indicates that the degree of linear correlation among the selected variables is weak. For most of the interactions the degree of linear correlation is significantly very weak.

**Table 1:** Correlation matrix.

Variable	*kFund*_*i*_	*noArt3*_*i-1*_	*avgIf3*_*i-1*_	*avgCit3*_*i-1*_	bc3*10 ^*2*^_*i*_	dc3*10 ^*2*^_*i*_	*cc3*_*i*_	ec3*10 ^*2*^_*i*_	*carAge*_*i*_
*kFund*_*i*_ *	1.0000								
*noArt3*_*i-1*_	0.3085	1.0000							
*avgIf3*_*i-1*_	0.0683	0.0425	1.0000						
*avgCit3*_*i-1*_	0.0598	0.0361	0.2894	1.0000					
bc3*10 ^*2*^_*i*_	0.1767	0.4591	0.0396	0.0243	1.0000				
dc3*10 ^*2*^_*i*_	0.0759	0.1365	0.1456	0.1098	0.0880	1.0000			
*cc3*_*i*_	-0.0894	-0.3570	0.0332	0.0454	-0.1760	0.0439	1.0000		
ec3*10 ^*2*^_*i*_	0.0099	0.0423	0.0474	0.0166	-0.0039	0.4248	0.0309	1.0000	
*carAge*_*i*_	0.1598	0.3239	0.0063	0.0544	0.1168	-0.0002	-0.2447	-0.0032	1.0000

* Note: *kFund* is the amount of funding divided by 1000.

The results of the multiple regression analysis are shown in Table 2. In the rest of this section we take each of the independent variables in turn and evaluate its effect on the amount of funding that researchers receive. According to the results, the rate and quality of the publications in the past three years have a positive impact on the amount of funding in the following year. Among them the number of publications has the highest impact while the effect of the average number of citations is the lowest. Hence, it can be said that researchers who are highly productive in terms of the quantity and quality of their papers receive on average higher amount of funding. It was argued in some studies (*e*.*g*. [24,43]) that higher amount of funding available will result in higher number of publications. Here we found that the other direction of the equation could be also true. This partially confirms the existence of the Matthew effect in the community of the NSERC funded researchers.

**Table 2:** Regression result.

*kFund*_*i*_	*Coef*.	*Std*. *Err*.	*t*	*P>|t|*	*[95% Conf*. *Interval]*	
*noArt3*_*i-1*_	5.548908***	.0961418	57.72	0.000	5.360471	5.737346
*avgIf3*_*i-1*_	3.659511***	.3372827	10.85	0.000	2.998438	4.320584
*avgCit3*_*i-1*_	.258806***	.0376524	6.87	0.000	.1850074	.3326046
bc3*10 ^*2*^_*i*_	45.58743***	4.312701	10.57	0.000	37.13455	54.04031
dc3*10 ^*2*^_*i*_	27.94798***	3.750963	7.45	0.000	20.59611	35.29986
*cc3*_*i*_	8.185887***	1.103446	7.42	0.000	6.023136	10.34864
ec3*10 ^*2*^_*i*_	-14.57707***	3.202601	-4.55	0.000	-20.85416	-8.299987
*careerAge*_*i*_	.8142736*	.4190712	1.94	0.052	-.0071046	1.635652
*careerAge* ^*2*^_*i*_	.057353**	.0263626	2.18	0.030	.0056824	.1090236
**Largest component dummy variable**						
*dInLargest*	15.3695***	.9099608	16.89	0.000	13.58598	17.15302
**Affiliations dummy variable**						
*dAcademia*	4.822487**	2.145102	2.25	0.025	.6180933	9.026881
**Provinces dummy variables**						
*dQuebec*	6.233359***	.9871349	6.31	0.000	4.298578	8.168141
*dBColumbia*	9.389953***	1.181226	7.95	0.000	7.074754	11.70515
*dAlberta*	-1.783301	1.26717	-1.41	0.159	-4.266951	.7003479
*dSaskatchewan*	-4.504571**	2.081214	-2.16	0.030	-8.583745	-.4253972
*dNBrunswick*	-6.073225**	2.560385	-2.37	0.018	-11.09157	-1.054878
*dManitoba*	-10.56926***	2.179204	-4.85	0.000	-14.8405	-6.298031
*dNFoundland*	-12.21***	2.819985	-4.33	0.000	-17.73717	-6.682842
*dPEdward*	-26.7532***	6.724452	-3.98	0.000	-39.9331	-13.57329
*dNScotia*	-5.472374***	1.904909	-2.87	0.004	-9.205989	-1.738759
*_cons*	15.83564***	2.621302	6.04	0.000	10.6979	20.97339

Notes:

* p<0.10,

** p<0.05,

*** p<0.01, number of observations: 72,267

Interestingly, the impact of average journal factor is much higher than the impact of the average number of citations. A possible explanation may be related to the reputation of researchers possibly affecting their success in publishing in high impact factor journals as we expect that scientists who are well known and more recognized within their scientific community have on average higher chance of publishing articles in higher quality journals. Therefore, having high average of journal impact factors of the journals a researcher is publishing in can also partially reflect the likelihood of the researcher being more reputable. This is also partially confirmed by the positive impact of the career age on the amount of funding that will be discussed later. In addition, it is quite in line with Arora and Gambardella [15] who suggest that well-known highly reputable researchers with an established record of successes are more likely to receive higher amount of funding. On the other hand, the average number of citations mainly reflects the scientific value and the credibility of a researcher’s publications within his/her community. According to the results, although higher quality works may result in higher amount of funding, it seems that funding is more biased toward the reputation of a researcher rather than just the scientific impact of his/her publications.

Network structure variables reflect the impact of collaboration patterns and researchers’ position in the co-authorship network on the amount of funding that they receive. According to Table 2, betweenness centrality (*bc*) has a significant positive impact on the amount of funding. A researcher with high betweenness centrality is playing an important role in the network as he/she is positioned on a relatively high proportion of shortest paths between other researchers. Hence, researchers would have to go through the researcher with high betweenness centrality to reach other researchers/communities. Therefore, these highly central researchers can control the flow of knowledge and can influence the formation and evolution of scientific teams and research projects acting as *gatekeepers*. Based on these explanations, the positive relation between funding and betweenness centrality was quite expected.

Degree centrality can be regarded as a proxy for the size of scientific team of researchers in the co-authorship network. In other words, a researcher with high degree centrality has on average higher number of co-authors in comparison with the counterparts with lower degree centrality. Therefore, they may have better access to other researchers that might enable them to get involved in more projects. Moreover, a researcher with high degree centrality can also be regarded as a *social* researcher who is in contact with a relatively high number of other researchers that might enable him/her to be aware of resource transactions among other researchers, hence increasing the chance of being involved in new projects and/or securing new funding resources. In contrast, a *peripheral* researcher has on average few or even no relations, which lowers his/her chances to meet other potential researchers, get involved in high priority well-defined projects, and secure new funding resources. Another advantage of a social researcher over a peripheral one is the better access to the knowledge resources that might enable him/her to come up with a higher variety and more interesting research ideas. This might also help the researcher to secure a higher amount of research funding, since the quality of the proposals is supposed to be one of the main factors for the funding allocation. According to Table 2, our results also suggest a positive impact of the degree centrality on the amount of funding that researchers receive.

As it can be seen in Table 2, clustering coefficient (cc) has also a positive impact on funding. As mentioned earlier, clustering coefficient is a measure of the number of triangles (cliques) in a network, and is also called the *cliquishness*. In the co-authorship network, a researcher with high clustering coefficient has on average a more connected neighborhood. If his/her neighborhood is fully connected (*i*.*e*. there exists a connection between each pair of the researchers in the neighborhood) then his/her clustering coefficient would be one. As the number of connections in the neighborhood decreases the value of the cliquishness gets closer to zero. The positive relation between cliquishness and funding shows the importance of being involved in well-connected communities. Apart from confirming the important positive role of direct connections (degree centrality), this result also suggests that being a member of a better connected community increases the chance of securing more money. A researcher in a more connected community is more likely to be involved in more multidisciplinary research which requires active interactions among all members of the team. Hence, our results partially suggest that working in a multidisciplinary project can also increase the chance of getting more money for the research. The complex nature of modern science forces researchers to go beyond the restricted circle of their direct connections and get involved in more interdisciplinary research. This allows them not only to get access to novel skills and enrich their own expertise but also to the new financial resources.

Eigenvector centrality is a more global network analysis measure since it considers the overall structure of the network. Based on our results a negative relation is observed between the eigenvector centrality of the researchers and the amount of funding (Table 2). Observing a negative impact of the eigenvector centrality along with the positive effect of the other examined network structure variables that were already discussed may indicate the importance of the direct connections in securing more money. According to the results, being directly connected to many researchers and working in relatively bigger teams increases the probability of success in obtaining research grants. Also, researchers involved in tight communities within highly clustered neighborhoods will more likely be able to assure that the information is transmitted through them and that they can access all the knowledge available in the neighborhood. On the other hand, if one is not directly involved within any tightly knit community but instead is only connected to highly important researchers he/she may not necessarily have the greatest local influence and may be in fact quite peripheral. Also, such researcher may have a quite limited brokering potential and may not be able to reach the knowledge available in other communities. Hence, researchers with many contacts who are tightly embedded within the dense network of research partnerships are more likely to get higher amount of funding than those who only have a few connections to highly central contacts. It seems that having few important friends does not really help in securing more funding.

Another interesting point is the higher intensity of the network structure variables in comparison with the other independent variables (Table 2). This highlights a greater importance of social and professional connections (networking in general) in securing more funding in comparison with some scientific and performance related indicators like past productivity and quality of the publications. This can be also regarded as an indicator of the review bias in NSERC funding allocation system as researchers who are involved in particular social networks are more likely to secure more funding (special thanks to the anonymous reviewer for bringing up this point). Building an active and effective collaboration network is thus more important than producing papers!

Evaluating the effect of the career age of researchers reveals the positive relation between the age and the amount of funding (Table 2). In general, as the career age of the researchers grows they gain more reputation in the scientific community. In addition, as they move forward they acquire more experience in writing funding proposals and searching for new funding resources. Moreover, their collaboration network becomes more connected gradually. Hence, older scientists tend to receive higher amount of funding. The small positive coefficient of quadratic term of the career age variable also confirms this finding.

The analysis of the largest component dummy variable (*dInLargest*) reveals that being in the largest component of the co-authorship network can be advantageous for a researcher in securing higher amount of funding. It is quite expected since being in the largest component means that such researcher is a part of a connected network hence he/she can potentially get access to more researchers and it would be more likely for him/her to secure more sources of funding than a researcher outside the largest component. This might also provide him/her with more information about research projects, new financial sources, research ideas, *etc*.

We also evaluated the effect of the institution type of researchers measured by *dAcademia* dummy variable. Our results suggest that academic researchers are significantly different from the non-academic ones and are on average more likely to receive higher amount of funding. This finding was quite expected as the number of the industry-related programs is relatively limited in NSERC. In addition, industrial researchers might have access to other internal financial resources hence applying less for the federal funding.

We checked for the impact of being located in different Canadian provinces by including the provinces dummy variables in the model. For this purpose we omitted Ontario and defined dummy variables for the remaining nine Canadian provinces to compare their impact with Ontario. All the provinces dummy variables were significant at the level of 95% except for Alberta. Interestingly, researchers who are located in Quebec and British Columbia tend to receive more amount of funding in comparison with the researchers of Ontario. However, the other researchers who are located in Saskatchewan, New Brunswick, Manitoba, Newfoundland and Labrador, Prince Edward, and Nova Scotia provinces are on average receiving lower amount of funding in comparison with their counterparts who are located in Ontario. This partially highlights the importance of the location factor in regard to the amount of funding that is allocated to the researchers. However, this is not surprising since most of the top ranking Canadian universities are located in Ontario, Quebec, British Columbia and Alberta.

## Conclusions

This paper analyzed the impact of various influential factors of different types on the amount of funding that researchers receive. We employed social network analysis and non-linear multiple regression model to assess the impact of the selected factors on funding at the individual level. To our knowledge this is the first comprehensive study that considers the network structure variables along with several other factors and evaluates their impact on researchers’ funding at the individual level.

Our findings showed the significant role of collaboration and networking in research activities. Collaboration can generate large advantages both for the researchers and the society. Through the collaborative scientific activities, different skills and ideas are combined, and resources (*e*.*g*. knowledge, information, financial resources, equipment, *etc*.) are thus used more efficiently. This can bring economies of scale in scientific activities and may avoid research duplication [28]. In addition, collaboration trains the available skills that will result in the development of new expertise [44] and better flow of information. Hence, being a member of a larger team may bring new opportunities to a researcher such as getting involved in new projects, exchanging knowledge with other skillful scientists, getting access to new funding resources, *etc*. This is in line with our results which suggest that in order to secure more funding a researcher should get involved with a lot of direct collaborators, *i*.*e*. he/she should become part of larger active teams. The sum of all the direct and indirect ties that a researcher possesses represents his/her social capital.

Moreover, we observed that getting well interconnected within tightly knit research communities enhances the funding chances as well. Such network neighborhoods are more cohesive and dense, and the ties can generally be considered of higher quality, because the dense and clustered relationships foster friendship and trust [45]. Clustering promotes community norms of collaboration, risk sharing and resource pooling among the members of the network, thereby leading to their improved creativity and enhanced system performance [46]. Also, the transmission of knowledge and information within the clustered neighborhoods is much more efficient [29]. Being a part of such tightly knit research community should thus allow the researchers to know more about potential research projects and funding opportunities within their community, and to get involved in them easier.

In addition, we found that brokering (gatekeeping) power in the network has a significant impact on the ability of researchers to attract funding. Occupying a network position which would allow researchers for some control over the flow of knowledge and information in the scientific community will not only enable the researchers to get access to more knowledge, but it would also open more funding opportunities for them. Even though *gatekeepers* may not necessarily have many contacts in the network their specific location between network neighborhoods makes them critical to collaboration across various teams, often from different institutions and/or scientific fields. Given the increasingly multidisciplinary nature of the science this is extremely important for many scientific projects today, and *gatekeepers* hence play a strategic role in creating connections among various scientific communities, projects, institutions or researchers. They may thus naturally end up being involved in many research grants. *Gatekeepers* seem to be great candidates for collaboration. If one gets connected to them it may not only enhance his/her ability to access knowledge or expertise from various scientific fields, but it may also improve the chances of success in acquiring research funding.

Surprisingly, we found that *diplomats*, *i*.*e*. scientists with many important and centrally positioned collaborators, are themselves not very successful in obtaining financial support for their research projects. We observed that connecting with *diplomats* might not be that helpful and it may even harm the researcher’s funding prospects. The *diplomats* may be connected to many important individuals but this does not necessarily mean that they have a strong influence in the community, that have a great access to knowledge or that they are able to shape the flow of knowledge in the network. Getting involved with important individuals without getting immersed in the research communities thus seems not to improve a researcher’s chances of funding success.

Analyzing the effect of the past productivity of researchers on funding revealed a positive relation between both quantity and quality of their papers on the amount of funding that they receive. Hence, as expected, more productive researchers are more likely to receive higher amount of funding. Interestingly, a much stronger effect was observed for the impact factor measure in comparison with the citation count indicator. Hence, it seems that having a paper of any quality published in a high impact factor journal improves the funding prospect much more than publishing a highly cited paper in an average journal. This suggests that the value of one’s work is nowadays assessed more by the reputation of the journal or of the researcher rather than by the actual contribution of the work to the knowledge advancement or its impact on the research community.

In addition, it was observed that as the career age of the researchers grows the amount of grants also increases. Therefore, it is more probable for the senior researchers to secure higher amount of funding in comparison with their junior counterparts. This finding was also quite expected since as the career age of the researchers grows they get on average more reputation in the scientific community that they work while their collaboration network also becomes more established. Moreover, senior researchers might be more experienced in writing funding proposals and applying for new grants.

Furthermore, we found that academic researchers are more likely to receive higher amount of funding rather than the researchers who are affiliated with non-academic environment. Industrial researchers usually have access to internal financial resources within their company and do not apply for the federal funding except for the specific projects designed to foster industry-academia collaboration. Finally, according to the results Canadian provinces can be divided into two groups namely, high and low funding provinces. Ontario, Quebec, British Columbia, and Alberta can be assigned to the high funding group of provinces where the researchers who are located in the mentioned group receive on average higher amount of funding. Within the high funding provinces, it was observed that researchers from Quebec and British Columbia are more likely to receive on average higher amount of funding than their counterparts in Ontario while no significant difference was observed for the amount of funding between the researchers in Ontario and Alberta. The other six Canadian provinces belong to the low funding group of provinces. Researchers located in the low funding provinces receive on average lower amount of funding from all the provinces in the high funding group.

As the main Canadian federal funding agency, NSERC aims to advance knowledge and foster research and development activities through allocating funding to the researchers via various funding programs. Our results are in line with NSERC goals confirming the value of the high quality research and the performance of the researchers in receiving more money. Hence, the funding seems to be allocated fairly, based on the merits measured by the quantity and quality of publications. However, since we found that funding is slightly biased toward senior researchers we support funding strategies providing young productive researchers with more opportunities which would enable them to show their capabilities. Moreover, it is proposed to reconsider the evaluation criteria of the reviewers and to put more value on the actual quality of the publications and their documented impact on research community, as opposed to the journal impact factors.

And what should we advise the researchers who want to increase the funding for their projects? Obviously, publish, and publish a lot; do not leave any original research findings unpublished. Never mind if your work is not cited, but target high impact factor journals for your publications. Focus on networking and collaboration, because how you build your network is more important than what you publish. Create many partnerships and select productive scientists to work with you. Get well interconnected within your research community, foster friendship and trust within your teams. Find influential researchers connected to various research groups, search for the ones who have control over the network and the flow of information. However, do not rely on solely connecting to highly prolific researchers, because making a few important friends will just not get you there!

## Limitations and Future Work

We were exposed to some limitations in this paper. First, we selected Scopus for gathering information about the NSERC funded researchers’ articles. Since Scopus and other similar databases are English biased non-English articles are underrepresented [47]. Secondly, since Scopus data were less complete before 1996, we chose the time interval of 1996 to 2010 for our analysis. Another inevitable limitation concerning the data was the spelling errors and missing values. Although Scopus is confirmed in the literature to have a good coverage of articles, as a future work it would be recommended to focus on other similar databases to compare and confirm the results.

Furthermore, we had some limitations in measuring scientific collaboration among the researchers as we were unable to capture other links that might exist among the researchers like informal relationships. These types of connections are never recorded and thus cannot be quantified, but there are certainly some knowledge exchanges occurring during such associations that could affect the network performance. In addition, there are also some drawbacks in using co-authorship as an indicator of scientific collaboration since collaboration does not necessarily result in a joint article [26]. An example could be the case when two scientists cooperate together on a research project and then decide to publish their results separately [25]. Hence, future work can address this issue by taking other types of collaboration networks into the consideration.

Moreover, there are many other variables which might have an impact on funding allocation decisions, but were not considered in this paper due to the limited data. For example, the quality of proposals is an important factor which we were not able to assess. As the objective of this research was to evaluate the impact of the quantitative factors on funding, quality of the research proposals/projects were beyond the scope of this study. However, future works can take this issue into consideration. Moreover, the conformity with the eligibility criteria may play an important role. For example, some of the NSERC programs focus mainly on their relevance to the industry or whether industrial funding is available in addition to the NSERC funding (*e*.*g*. NSERC IRDF program). Another example is training of highly qualified personnel, *i*.*e*. the number of students that the researcher has supervised and whether these students have ended up in qualified jobs after leaving the university, which is also considered in the funding allocation decision. We were unable to access such information.

Last but not least, this study focused on the main Canadian federal funding agency. One of the reasons of such funding source selection was its large coverage as we realized that until recently almost all the Canadian researchers active in natural sciences and engineering have been being funded by NSERC, mainly through the Discovery Grants Program [16]. Although this coverage partially weakens the need for the control group of the non-funded researchers, lack of information on researchers who were unsuccessful in receiving funding was another limitation of the study as such data was not available to the public. We believe results can be generalized to other agencies that are active in supporting research in social sciences and engineering. To complement and confirm the findings of this research in other fields it would be suggested to focus on other Canadian funding organizations (*e*.*g*. SSHRC and CIHR) as the scope and regulations of the funding programs as well as the nature of the research in different fields might affect the results. In addition, research and scientific collaboration knows no borders thus analyzing the inter-relations among funding and influencing factors in other countries can be also informative. This might help researchers as a complementary guide in securing international funding.

## Supporting Information

S1 Table List of abbreviations and acronyms. (DOCX)

## References

[1] Griffith R., Redding S., & Van Reenen J. Mapping the two faces of R&D: Productivity growth in a panel of OECD industries. Review of Economics and Statistics 2004; 86(4), 883–895.

[2] Klette T. J., & Kortum S. Innovating firms and aggregate innovation (No. w8819). National Bureau of Economic Research 2002.

[3] Oyo, B., Williams, D., & Barendsen, E. A system dynamics tool for higher education funding and quality policy analysis. Proceedings of the 24th International Conference of the System Dynamics Society 2008.

[4] Martin B. R. The changing social contract for science and the evolution of the university Science and Innovation: Rethinking the Rationales for Funding and Governance. Edward Elgar 2003, Cheltenham, 7–29.

[5] Jacob B. A., & Lefgren L. The impact of research grant funding on scientific productivity. Journal of Public Economics 2011; 95(9), 1168–1177. 21857758 10.1016/j.jpubeco.2011.05.005 PMC3156466

[6] Braun D. Lasting tensions in research policy-making—a delegation problem. Science and Public Policy 2003; 30(5), 309–321.

[7] Leydesdorff L., & Wagner C. Macro-level indicators of the relations between research funding and research output. Journal of Informetrics 2009; 3(4), 353–362.

[8] Elzinga A., & Jamison A. Changing policy agendas in science and technology Handbook of Science and Technology Studies Ed. by Sheila Jasanoff et al (London: Sage) 1995.

[9] Sanz Menéndez L., & Borrás S. Explaining changes and continuity in EU technology policy: The politics of ideas. Unidad de Políticas Comparadas (CSIC) 2000; Working Paper 00–01.

[10] Rip A. The republic of science in the 1990s. Higher Education 1994; 28(1), 3–23.

[11] Irvine J., & Martin B. R. Foresight in science: Picking the winners. Pinter London 1984.

[12] Polster C. The nature and implications of the growing importance of research grants to Canadian universities and academics. Higher Education 2007; 53(5), 599–622.

[13] Geuna A. The changing rationale for European university research funding: Are there negative unintended consequences? Journal of Economic Issues 2001; 607–632.

[14] Industry Canada. Achieving excellence: Investing in people, knowledge and opportunity. Ottawa: Government of Canada, Canada's Innovation Strategy. Industry Canada.Gc.Ca 2002 (Accessed on January 22, 2004).

[15] Arora A., & Gambardella A. The impact of NSF support for basic research in economics. Annales d'Economie et de Statistique 1998; 91–117.

[16] Godin B. The impact of research grants on the productivity and quality of scientific research. No. 2003. INRS Working Paper 2003.

[17] Payne A. A., & Siow A. Does federal research funding increase university research output? Advances in Economic Analysis & Policy 2003; 3(1).

[18] Shapira P., & Wang J. Follow the money. Nature 2010; 468(7324), 627–628. doi: 10.1038/468627a 21124430 10.1038/468627a

[19] Heffner A. G. Funded research, multiple authorship, and subauthorship collaboration in four disciplines. Scientometrics 1981; 3(1), 5–12.

[20] Adams J. D., Black G. C., Clemmons J. R., & Stephan P. E. Scientific teams and institutional collaborations: Evidence from US universities, 1981–1999. Research Policy 2005; 34(3), 259–285.

[21] Defazio D., Lockett A., & Wright M. Funding incentives, collaborative dynamics and scientific productivity: Evidence from the EU framework program. Research Policy 2009; 38(2), 293–305.

[22] Ebadi A., & Schiffauerova A. How to Become an Important Player in Scientific Collaboration Networks? Submitted to the Journal of Informetrics 2015.

[23] Gladwell M., Outliers: The story of success 2008.

[24] Beaudry C., & Allaoui S. Impact of public and private research funding on scientific production: The case of nanotechnology. Research Policy 2012; 41(9), 1589–1606.

[25] Katz J. S., & Martin B. R. What is research collaboration? Research Policy 1997; 26(1), 1–18.

[26] Tijssen R. J. Is the commercialisation of scientific research affecting the production of public knowledge? Global trends in the output of corporate research articles. Research Policy 2004; 33(5), 709–733.

[27] De Solla Price D. Big science, little science. Columbia University 1963; New York, 119–119.

[28] Ubfal D., & Maffioli A. The impact of funding on research collaboration: Evidence from a developing country. Research Policy 2011; 40(9), 1269–1279.

[29] Schilling M. A., & Phelps C. C. Interfirm collaboration networks: The impact of large-scale network structure on firm innovation. Management Science 2007; 53(7), 1113–1126.

[30] Gulati R., & Gargiulo M. (1999). Where Do Interorganizational Networks Come From? *American Journal of Sociology*, 104, 177–231.

[31] Stuart T. E. (2000). Interorganizational alliances and the performance of firms: A study of growth and innovation rates in a high-technology industry. *Strategic management journal*, 21(8), 791–811.

[32] Borgatti S. P. Centrality and network flow. Social Networks 2005; 27(1), 55–71.

[33] Wasserman S. Social network analysis: Methods and applications. Cambridge University Press 1994.

[34] Bonacich P. Factoring and weighting approaches to status scores and clique identification. Journal of Mathematical Sociology 1972; 2(1), 113–120.

[35] Hanneman R. A., & Riddle M. Concepts and measures for basic network analysis The Sage Handbook of Social Network Analysis 2011; SAGE Publications Ltd, 340–369.

[36] Watts D. J., & Strogatz S. H. Collective dynamics of ‘small-world’ networks. Nature 1998; 393(6684), 440–442. 9623998 10.1038/30918

[37] Merton R. K. The sociology of science: Theoretical and empirical investigations. University of Chicago press 1973.

[38] Kyvik S., & Olsen T. B. Does the aging of tenured academic staff affect the research performance of universities? Scientometrics 2008; 76(3), 439–455.

[39] Moed H. F., Van Leeuwen T. N., & Reedijk J. A critical analysis of the journal impact factors of Angewandte chemie and the journal of the American chemical society inaccuracies in published impact factors based on overall citations only. Scientometrics 1996; 37(1), 105–116.

[40] Seglen P. O. Why the impact factor of journals should not be used for evaluating research. BMJ (Clinical Research Ed.) 1997; 314(7079), 498–502. 10.1136/bmj.314.7079.497 PMC2126010 9056804

[41] Seglen P. O. The skewness of science. Journal of the American Society for Information Science 1992; 43(9), 628–638.

[42] Fortin J. M., & Currie D. J. (2013). Big science vs. little science: how scientific impact scales with funding. *PloS one*, 8(6), e65263. 23840323 10.1371/journal.pone.0065263 PMC3686789

[43] Arora A., David P. A., & Gambardella A. Reputation and competence in publicly funded science: Estimating the effects on research group productivity The Economics and Econometrics of Innovation 2000; (pp. 141–176) Springer.

[44] Lee S., & Bozeman B. The impact of research collaboration on scientific productivity. Social Studies of Science 2005; 35(5), 673–702.

[45] Burt D. R. Bandwidth and echo: Trust, information, and gossip in social networks In: Casella A. & Rauch J.E. (Eds.), Networks and Markets: Contributions from Economics and Sociology, New York: Russel Sage Foundation 2001.

[46] Uzzi B. and Spiro J. Collaboration and creativity: The small world problem. American Journal of Sociology 2005; 111(2), 447–504.

[47] Okubo Y. Bibliometric indicators and analysis of research systems: Methods and examples. No. 1997/1. OECD Publishing 1997.

