# Supplementary material for: How to Receive More Funding for Your Research? Get Connected to the Right People!
Source: PLoS One. 2015 Jul 29;10(7):e0133061. doi: 10.1371/journal.pone.0133061 (PMC4519253; doi:10.1371/journal.pone.0133061)
Supplement: S1 Table — (DOCX) [file pone.0133061.s001.docx]

**S1 Table.** List of abbreviations and acronyms

| ***Abbreviation*** | ***Meaning*** |
| --- | --- |
| *NSERC* | Natural Sciences and Engineering Research Council |
| *SSHRC* | Social Sciences and Humanities Research Council |
| *CIHR* | Canadian Institutes of Health Research |
| *R&D* | Research and Development |
| *IRDF* | Industrial R&D Fellowships |
| *BC* | Betweenness Centrality |
| *CC* | Clustering Coefficient |
| *DC* | Degree Centrality |
| *EC* | Eigenvector Centrality |
